# The SOS response increases bacterial fitness, but not evolvability, under a sublethal dose of antibiotic

**DOI:** 10.1098/rspb.2015.0885

**Published:** 2015-10-07

**Authors:** Clara Torres-Barceló, Mila Kojadinovic, Richard Moxon, R. Craig MacLean

**Affiliations:** 1 Department of Zoology, University of Oxford, Oxford OX1 3PS, UK; 2 Institut des Sciences de l'Evolution, CNRS-Université Montpellier 2, Montpellier, France; 3 University of Oxford Medical Sciences Division, John Radcliffe Hospital, Oxford OX3 9DS, UK

**Keywords:** bacteria, stress, SOS response, fitness, evolvability, antibiotics

## Abstract

Exposure to antibiotics induces the expression of mutagenic bacterial stress–response pathways, but the evolutionary benefits of these responses remain unclear. One possibility is that stress–response pathways provide a short-term advantage by protecting bacteria against the toxic effects of antibiotics. Second, it is possible that stress-induced mutagenesis provides a long-term advantage by accelerating the evolution of resistance. Here, we directly measure the contribution of the *Pseudomonas aeruginosa* SOS pathway to bacterial fitness and evolvability in the presence of sublethal doses of ciprofloxacin. Using short-term competition experiments, we demonstrate that the SOS pathway increases competitive fitness in the presence of ciprofloxacin. Continued exposure to ciprofloxacin results in the rapid evolution of increased fitness and antibiotic resistance, but we find no evidence that SOS-induced mutagenesis accelerates the rate of adaptation to ciprofloxacin during a 200 generation selection experiment. Intriguingly, we find that the expression of the SOS pathway decreases during adaptation to ciprofloxacin, and this helps to explain why this pathway does not increase long-term evolvability. Furthermore, we argue that the SOS pathway fails to accelerate adaptation to ciprofloxacin because the modest increase in the mutation rate associated with SOS mutagenesis is offset by a decrease in the effective strength of selection for increased resistance at a population level. Our findings suggest that the primary evolutionary benefit of the SOS response is to increase bacterial competitive ability, and that stress-induced mutagenesis is an unwanted side effect, and not a selected attribute, of this pathway.

## 1. Introduction

Exposure to stress, which we define as any environmental perturbation that decreases bacterial growth rate or survival, induces the expression of stress–response pathways that can result in profound changes in bacterial gene expression and physiology [1,2]. Intriguingly, stress–response pathways are often associated with the expression of error-prone, translesion synthesis DNA-polymerase enzymes that elevate the mutation rate under stress [3,4]. However, the underlying evolutionary processes that couple stress to elevated mutagenesis remain unclear. One hypothesis is that stress-induced DNA polymerases directly increase bacterial fitness by increasing protection against stress, for example, by rescuing cells from replication arrests that occur when DNA lesions take place [5]. In support of this idea, knocking out genes involved in stress-induced mutagenesis has been shown to decrease bacterial survival in the presence of DNA-damaging agents [6,7]. According to this hypothesis, the primary function of stress-induced polymerases is to increase survival under stress and increased mutagenesis is simply a secondary consequence of the primary roles of these enzymes [8,9]. Alternatively, it has been argued that these specialized enzymes exist primarily because they constitute an evolved mechanism to transiently elevate the mutation rate to generate a larger pool of mutants in times of stress [5,10]. According to this hypothesis, stress–response pathways are associated with an elevated mutation rate because natural selection drives the evolution of mechanisms that couple the evolutionary demand for innovation imposed by stress to the supply of evolutionary novelty provided by mutation [10]. For example, it has been argued that stress-induced polymerases increase the ability of *Escherichia coli* to adapt to long-term nutrient starvation [11] and antibiotics [12,13].

Although the existing literature suggests that stress–response pathways may increase evolvability and competitive ability, direct and quantitative estimates of the effect of stress–responses on fitness are currently lacking. Thus, the central and unresolved problem in our understanding of the evolutionary biology of stress–responses is to understand the relative importance of the short and long-term fitness benefits associated with stress–response pathways [4]. To tackle this problem, we used a simple and well-characterized model system involving the SOS response to antibiotic-induced DNA damage in the pathogenic bacterium *Pseudomonas aeruginosa* [14]. The SOS response is a classic bacterial stress–response pathway that is induced by DNA damage caused by a wide range of stressors, including antibiotics [12]. The SOS response is initiated when RecA binds to damaged ssDNA and induces the autocatalytic cleavage of the LexA repressor [15], resulting in the de-repression of genes involved in the SOS response. The SOS response is widespread across bacteria, but the sequence of the LexA binding site and the genes induced by the response vary considerably, even among closely related bacteria [16,17]. In *P. aeruginosa*, LexA regulates the expression of 15 genes [14,17], including genes involved in DNA repair (*recX* and *recN*), three low-fidelity, non-essential DNA polymerases (*dnaE2*, *imuB* and *imuA*) and an inhibitor of cell division, s*ulA*, that causes cell filamentation [12,18,19]. Despite the apparent simplicity of this pathway, previous work has shown that the dynamics of expression of this response are complex, involving several levels of regulation, peaks of activation, time frames and between-cell heterogeneity [15,20–22].

In this paper, we measure the impact of the SOS response on competitive fitness and evolvability in the presence of the fluoroquinolone antibiotic ciprofloxacin. Fluoroquinolones are potent, bactericidal antibiotics that inhibit DNA gyrase, which eventually leads to the formation of double stranded DNA breaks, stalled DNA replication forks and, ultimately, cell death [23,24]. Because fluoroquinolones cause the formation of DNA breaks they are potent inducers of the SOS response, suggesting that SOS-induced mutagenesis may catalyse the evolution of resistance to fluoroquinolones. To directly test this idea, we measured the competitive fitness and evolvability of an SOS proficient wild-type strain of *P. aeruginosa* relative to an isogenic SOS uninducible strain carrying a mutation in *LexA* (S125A) that blocks the induction of the SOS response by preventing autocatalytic cleavage of LexA dimers, the repressor associated to the SOS regulon [14].

Previous studies that have investigated the evolutionary benefits of SOS expression have tended to focus on situations where bacterial populations are challenged with a lethal dose of antibiotic (>minimum inhibitory concentration, MIC). Under these conditions, selection for resistance is so strong that it is very difficult to distinguish if benefits of the SOS pathway stem from increased survival or evolvability [12,13]. There is growing recognition that sublethal doses of antibiotic may play an important role in the evolution of resistance [25,26] and recent work has shown that exposure to sublethal doses of antibiotics leads to stress-induced mutagenesis [27]. We focused on studying the evolutionary benefits of SOS expression under a moderate dose of ciprofloxacin that leads to 50% mortality; crucially, this dose of ciprofloxacin leads to maximal induction of the SOS response.

## 2. Material and methods

### (a) Bacterial strains and media

Wild-type *P. aeruginosa* PAO1 strain (WT), a WT gentamicin-resistant strain (WT-GmR) and an isogenic mutant carrying mutation S125A in the *lexA* gene (LexA) were kindly provided by the laboratory of Dr F. Romesberg (Scripps Research Institute, USA); details of the construction of the mutant are provided in [14]. For competition and expression of the SOS response experiments we generated yellow fluorescent protein (YFP)-tagged WT strain and WTpLex : Lux as described in the electronic supplementary material. Four strains carry a gentamicin-resistance marker that is integrated downstream of *lexA* (WT and LexA strains) or at the *att*Tn7 insertion site (WT-YFP, WTpLexLux). Bacteria were cultured in M9KB broth supplemented at varying concentrations of ciprofloxacin (Sigma-Aldrich) as required. Cultures were incubated at 37°C without shaking or with orbital shaking at 200 r.p.m.

### (b) Measurement of SOS pathway expression and bacterial population density in the presence of ciprofloxacin

To measure the impact of ciprofloxacin exposure on SOS pathway expression, we measured luminescence output of the WTpLex : Lux strain across a gradient of ciprofloxacin relative to a WT control. We streaked glycerol stocks of the two strains out on M9KB plates and then inoculated 30 independent colonies of each strain into 200 µl of M9KB medium. After overnight incubation, cultures were diluted 100-fold into 200 µl of fresh M9KB medium that was supplemented with ciprofloxacin between 0 and 72 µg l^−1^. To assay the expression of the SOS pathway, we measured the luminescence output of diluted samples of mid-exponential phase cultures using a BMG Optima microtiter plate reader. To standardize measurements of SOS expression to viable cell density, we measured the viable cell titre of cultures by using Bactitre-Glo (Promega, Maddisson, WI, USA) to destructively measure the total cellular ATP content of samples that were used for SOS expression measurements (see electronic supplementary material, Material and methods for further details). Estimates of SOS expression were standardized relative to the mean value of WTpLex : Lux grown in the absence of ciprofloxacin.

To measure the impact on ciprofloxacin on bacterial population growth we did as explained in the electronic supplementary material.

### (c) Competition experiments

To measure how the SOS pathway influences competitive fitness, we directly competed the WT and LexA strains against each other, taking advantage of the fact that the LexA strain contains a gentamicin resistance cassette that was inserted into the PAO1 chromosome during construction of the LexA mutant [14]. First, we streaked out the WT and LexA strains from glycerol stocks on M9KB agar plates. Isolated colonies of each strain were then inoculated into M9KB broth used to set up pre-cultures. Cultures of each strain at exponential phase were combined, mixed and appropriate dilutions plated in M9KB with and without gentamicin (15 mg ml^−1^). Five independent cultures per four combinations (initial frequency of WT: 70, 50, 40 and 20%) were transferred in a 10^2^ dilution into a microcosm with fresh media plus 48 µg l^−1^ ciprofloxacin or not. After overnight growth, cultures were diluted and plated onto M9KB plates with and without gentamicin. Fitness of wild-type was calculated as the ratio of wild-type to LexA malthusian parameters [28]. To analyse the data from this experiment, we used a fully factorial ANOVA containing dose (0, 48 µg l^−1^), initial frequency of WT (70, 50, 40 and 20%) and genotype (WT, LexA) as explanatory factors. Where appropriate, analyses were carried out separately to compare genotypes for each dose.

To control for the possibly confounding effects of the gentamicin-resistance cassette inserted in the LexA strain, we carried out a control competition experiment between WT and WT-gmR, a mutant constructed from WT containing the same gentamicin-resistance cassette found in the LexA strain. Using the methods outlined above to carry out competitions, we found that the gentamicin-resistance cassette did not have any effect on fitness in the presence (*t*_11_ = −0.16, *p* = 0.878) or absence (*t*_11_ = 0.3191, *p* = 0.7623) of ciprofloxacin.

### (d) Mutation rate estimation

Mutation rate was calculated using a fluctuation test as described previously [29,30]. We tested the effect of a IC50 dose of ciprofloxacin (48 µg l^−1^) on mutation rate in eight independent cultures per strain (WT, LexA). We set up the cultures from individual colonies and cultured them with and without the antibiotic overnight. After that, we spread the bacteria directly into M9KB agar plates with the antibiotic Rifampicin (100 µg ml^−1^), incubated at 37°C for 24 h and counted resistant colonies. Appropriate dilutions were plated onto M9KB agar plates to count the total number of cells of the same cultures. Estimation of mutation rate values and confidence intervals (CIs) was done using Ma-Sandri-Sarkar Maximum (MSS) maximum-likelihood method as implemented in www.mitochondria.org/protocols/FALCOR.html [31]. Note that FALCOR calculates frequency, providing statistical interpretation of the data through overlapping 83% CIs about the median as recommended in [32].

### (e) Long-term evolution

To set up the long-term evolution experiment, we streaked out −80C glycerol stocks of the WT and LexA strains. After overnight incubation, we used isolated colonies to inoculate 48 independent M9KB cultures of each strain on a 96-well microtitre plate containing 200 µl of M9KB in each well. After overnight incubation, 100 µl of each initial pre-culture population was then frozen in 25% glycerol at −80°C. We transferred 1 µl from the remaining volume of each culture to a fresh 96-well microtiter plate that either contained culture medium lacking ciprofloxacin (*n* = 24 replicate populations strain^−1^) or contained ciprofloxacin at a concentration of 48 µg l^−1^ (*n* = 24 replicate populations strain^−1^). In other words, we set up a selection experiment consisting of four treatments (two strains and two antibiotic doses), each with 24 truly independent populations. Evolving populations were transferred to fresh media every day for a further 24 days and we have periodically frozen down 100 µl samples of evolving population in 25% glycerol at −80°C. To check for contamination, we sequenced the *lexA* and gentamicin-resistance cassette in all 40 of the populations used for fitness assays as described in the electronic supplementary material.

To measure the evolvability, we measured the rate of evolution of fitness through time in the selection experiment. For each treatment, we analysed the fitness of 10 replicate populations from four time points (transfers 7, 12, 17 and 23). We did not assay populations from the 60 ‘edge’ wells of the microtiter plate, as increased evaporation leads to elevated antibiotic concentrations in these populations. To be able to measure the fitness of ciprofloxacin-treated populations, the WT-YFP strain had to be pre-cultured 1 day earlier at 48 µg l^−1^ of the antibiotic, then mixed at 1 : 1 ratio and competitions were analysed after 6 h only. Fitness was determined using flow cytometry to measure the proportion of YFP tagged and untagged cells in each competition, as described in the electronic supplementary material. This allowed us to scale up our fitness assay and gave equivalent fitness estimates to estimating fitness by plating out colonies overnight.

Values of fitness are represented as relative to the ancestral WT fitness level. To test for differences in evolvability between the two genotypes (fitness through time), we analysed our complete fitness dataset using linear mixed-effects models (LME). In this model, fitness was the response value and genotype, antibiotic and time were included as a fixed categorical predictors. Note that time was not considered as a continuous variable as we only sampled four time points. To account for the non-independence between replicate populations and successive sampling in time, replicate was included as a random component of the model. In mixed models, model fit was checked by visual inspection of the residuals.

We also wanted to know if the levels of antibiotic resistance acquired by *P. aeruginosa* were affected by the presence of the SOS response, in the short- and the long term. To do so, we challenged initial (ancestral) and final (transfer 23) populations of the WT and LexA strains with increasing (twofold) concentrations of ciprofloxacin (minimum of 50 µg l^−1^ and maximum of 1600 µg l^−1^) and determined the MIC. The analysis was performed by measuring the optical density at 600 nm (OD_600_) in a spectrophotometer (BioTek Synergy 2) and recording the minimum antibiotic concentration at which the average of the populations were not able to produce any growth (OD_600_ < 0.1) after 24 h. We analysed MIC differences with a non-parametric Kruskal–Wallis test, all data together, or in pairs when considered interesting and correcting *p*-values with the Bonferroni correction method for multiple comparisons.

### (f) SOS response expression and evolution

To study the evolution of SOS pathway expression, we set up a second selection experiment in which the WTpLex : Lux strain was challenged with adapting to medium containing ciprofloxacin (48 µg l^−1^) or medium lacking antibiotics. The methods used to set up the experiment and propagate cultures are as described for the main selection experiment above. To measure how SOS expression evolves, we first transferred 1 µl aliquots of −80°C glycerol stocks into culture medium lacking antibiotics and these pre-cultures were incubated overnight at 37°C with continuous shaking. Pre-cultures of populations that evolved with ciprofloxacin were then diluted 200-fold into fresh medium containing ciprofloxacin and populations that evolved without ciprofloxacin were diluted 200-fold into fresh medium lacking ciprofloxacin. We measured SOS expression as described above. To test for the differences in the SOS expression, we used a linear model (LM) that considers time and the non-independence between replicate populations measured at different time points.

## 3. Results

### (a) SOS expression peaks at an intermediate dose of ciprofloxacin

**Figure 1.**
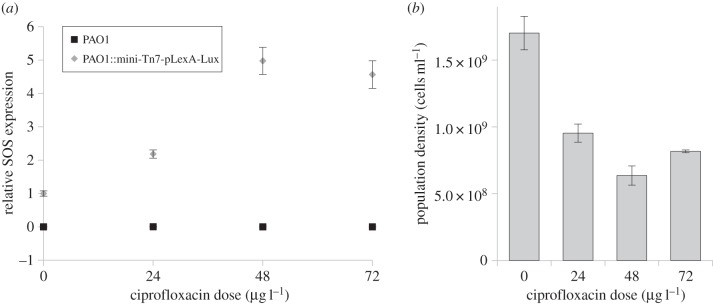
Impacts of ciprofloxacin on bacterial population density and SOS expression. Plotted points in (*a*) show the mean relative expression (±s.e.m.; *n* ≥ 3) of a bioluminescent SOS reporter construct across a gradient of ciprofloxacin in a WTpLex : Lux reporter strain (grey diamond) and a WT control lacking the reporter construct (black square). (*b*) The mean (±s.e.m.; *n* ≥ 3) density of viable cells in cultures of the WT strain that were grown overnight across a gradient of ciprofloxacin.

To investigate how ciprofloxacin exposure influences SOS expression, we measured the expression of a LexA-regulated luciferase reporter construct across a gradient of sub-MIC doses of ciprofloxacin. As expected, we found that SOS expression increases with ciprofloxacin dose (figure 1*a*), and we observed high induction (approx. fivefold) of SOS expression at an intermediate dose of ciprofloxacin (48 µg l^−1^). This dose causes an approximately twofold decrease in the viable cell density of overnight cultures (figure 1*b*). Because of the high expression of the SOS response and relatively mild effect on bacterial viability, we chose this sub-MIC dose of the antibiotic for the subsequent experiments.

### (b) The SOS pathway generates a short-term benefit in the presence of ciprofloxacin

**Figure 2.**
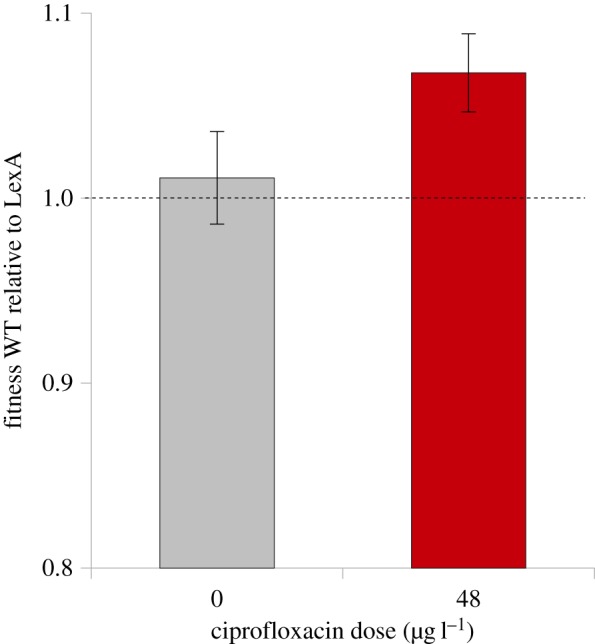
Short-term fitness effects of the SOS response. Competitive fitness of the WT strain relative to the LexA mutant, as measured using overnight competition experiments. Values are the average of 20 independent replicates starting from different WT : LexA frequencies and error bars are s.e.m. The dashed line represents no relative fitness difference (value of 1).

To test the hypothesis that the SOS pathway generates a direct fitness benefit under stress, we competed an SOS proficient strain (WT) against an isogenic LexA mutant that is incapable of inducing the SOS response in the presence of ciprofloxacin using an overnight competition assay. One important aspect of the design of this experiment is that we measured fitness in a multi-generational fitness assay. It is, therefore, possible that this assay measures the impact of the SOS pathway on both bacterial competitive ability and short-term evolvability. To account for this possibility, we manipulated the initial frequency of the wild-type strain in the competition assay between 20 and 70%. If the benefit of the SOS pathway in this assay stems from increased evolvability owing to stress-induced mutagenesis, then the WT strain should have high fitness when it is initially common, as the absolute number of beneficial mutations produced by SOS mutagenesis during the competition assay should increase with the frequency of the WT strain [33]. We found that the SOS pathway increased fitness by 6.78% in the presence of ciprofloxacin (figure 2; *t*_19_ = −3.22, *p* = 0.005), and this benefit was independent of the initial frequency of the WT strain (electronic supplementary material, figure S1; *F*_3,16_ = 1.18, *p* = 0.350). Although the absolute magnitude of this difference in fitness is small, this difference is sufficiently large for the WT strain to rapidly eliminate the LexA mutant strain in co-culture competition experiments that extend beyond 1 day (electronic supplementary material, figure S2).

Previous work [21] and our expression measurements (figure 1*a*) show that the SOS pathway is expressed at relatively low levels in the absence of any exogenous stressor, suggesting that the pathway may carry a fitness cost in the absence of stress. However, we found that the LexA mutant had indistinguishable competitive fitness to the WT strain in culture medium lacking ciprofloxacin (figure 2; mean fitness = 0.989; *t*_19_ = −0.44, *p* = 0.663).

The simplest explanation for why the SOS pathway is beneficial in the presence of ciprofloxacin is that the SOS-induced expression of genes involved in DNA replication and repair protects cells against the lethal effects of ciprofloxacin. However, we found that the minimal inhibitory concentration of the WT strain and LexA mutant were indistinguishable (MIC = 200 µg l^−1^, Kruskal–Wallis *χ*^2^_1_ = 2.11, *p* = 0.146).

### (c) The SOS pathway elevates the mutation rate under stress

**Figure 3.**
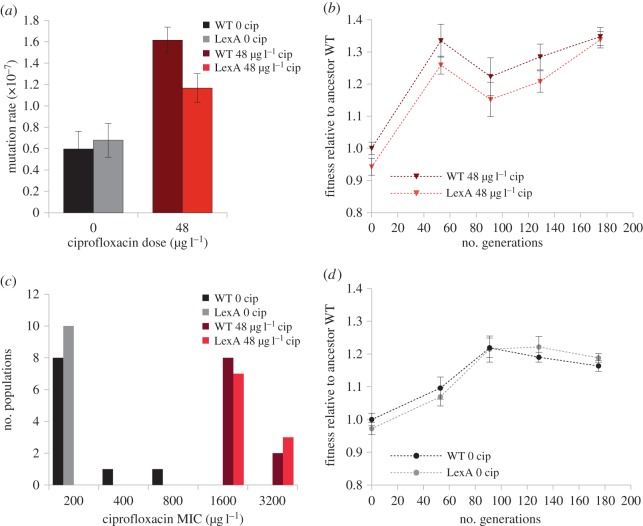
The impact of the SOS response on evolvability. (*a*) Mutation rate of WT and LexA in the absence or presence of ciprofloxacin, as calculated using a fluctuation test that measures the rate of mutation to rifampicin resistance. Estimation of mutation rate values (and 83% CIs) was done using MSS maximum-likelihood method from eight independent replicates. (*b*) The fitness trajectory of WT and LexA populations that evolved in the presence of ciprofloxacin. Fitness was measured relative to the ancestral WT strain, and plotted points show the mean and standard error of 10 replication populations for each treatment. (*c*) The distribution of MIC scores (ciprofloxacin concentration) in WT and LexA populations that were allowed to evolve for 200 generations in the presence and absence of ciprofloxacin. The ancestral WT and LexA clones have an MIC of 200 µg l^−1^. (*d*) The fitness trajectory of WT and LexA populations that evolved in the absence of ciprofloxacin. Fitness was measured relative to the ancestral WT strain, and plotted points show the mean and standard error of 10 replication populations for each treatment.

Previous work has shown that the *P. aeruginosa* SOS response pathway includes three non-essential polymerases, including DNAE2, an alternative and error-prone catalytic sub-unit of DNA-polymerase [14], suggesting that induction of this pathway should increase the mutation rate. To confirm that the SOS pathway is mutagenic, we measured the mutation rate of WT and LexA in the presence and absence of ciprofloxacin. The mutation rate did not differ between the WT and LexA strains in the absence of antibiotics (0.597 ± 0.166 × 10^−7^ and 0.679 ± 0.160 × 10^−7^ mutations per generation, respectively; figure 3*a*), demonstrating that there are no inherent differences in mutation rate between these strains. Adding ciprofloxacin resulted in an overall increase in the mean mutation rate (0.638 ± 0.041 × 10^−7^and 1.391 ± 0.224 × 10^−7^ mutations per generation for untreated and treated mean mutation rates, respectively; figure 3*a*). The mechanisms underpinning this are unclear, but exposure to ciprofloxacin must increase the mutation rate as a result of increased DNA damage [34], and it is also possible that the expression of error-prone polymerases that are regulated by alternative stress–response pathways [27], such as dinB [17], also contributes to the increased mutation rate. However, we found that exposure to the antibiotic increases the mutation rate more in the WT strain than in the LexA mutant, which directly demonstrates that the ciprofloxacin induced expression of the SOS pathway increases mutagenesis (1.615 ± 0.121 × 10^−7^and 1.168 ± 0.135 × 10^−7^ mutations per generation, respectively, figure 3*a*).

### (d) The SOS pathway does not accelerate the evolution of ciprofloxacin resistance

To test the hypothesis that the SOS pathway increases evolvability, we measured the rate of adaptation in populations founded by the WT and LexA strains that were allowed to evolve in culture medium containing ciprofloxacin (48 µg l^−1^) for 190 generations. To measure the rate of adaptation, we then determined the competitive fitness of clones sampled from the selection experiment in culture medium containing ciprofloxacin (figure 3*b*). Populations derived from the WT and LexA strains rapidly adapted to culture medium containing ciprofloxacin (LME model for antibiotic *t*_99_ = 101.31, *p* < 0.001) and average fitness increased by 35% over the course of the selection experiment (figure 3*b*). However, we found that the SOS pathway did not increase evolvability, as demonstrated by the fact that the average fitness of populations descended from the LexA and WT ancestral strains rapidly converged (LME model for genotype *t*_91_ = 0.11, *p* = 0.742; interaction time × genotype *t*_91_ = 0.01, *p* = 0.917). Given that the fitness of the ancestral LexA strain was 8% lower than that of the WT strain at the beginning of the experiment, these results imply that LexA populations actually evolved more rapidly at the outset of the experiment than the wild-type populations.

Because the dose of ciprofloxacin used in this experiment was sufficient to reduce live cell density by greater than twofold, increased ciprofloxacin resistance is likely to have been an important target for selection. As an alternative approach to measuring evolvability, we determined the ciprofloxacin MIC of the evolved clones sampled from the end of the experiment to determine the rate of evolution of resistance. All of the evolved clones had elevated ciprofloxacin resistance, and average MIC increased eightfold from 200 to 1600 µg l^−1^ (Kruskal–Wallis *χ*^2^_1_ = 34.24, *p* < 0.001). Crucially, ciprofloxacin resistance did not differ between the WT and LexA evolved clones (Kruskal–Wallis *χ*^2^_1_ = 0.25, *p* = 0.615; figure 3*c*).

As a control, we also measured the evolvability of the WT and LexA strains in culture medium lacking ciprofloxacin. Populations successfully adapted to the ciprofloxacin-free culture medium (*t*_94_ = 6.38, *p* < 0.001), but the increase in average fitness (17%) was lower than when *Pseudomonas* was challenged with adapting to ciprofloxacin (figure 3*d*). We did not find any difference in the rate of adaptation to culture medium between the WT and LexA populations (LM model for interaction time × genotype *t*_95_ = −1.19, *p* = 0.236; figure 3*d*), which demonstrates that there are no intrinsic differences in evolvability between these two strains in the absence of SOS induction. We found that the ciprofloxacin MIC of populations that evolved in the absence of ciprofloxacin did not increase.

### (e) Adaptation to stress decreases the expression of the SOS response

**Figure 4.**
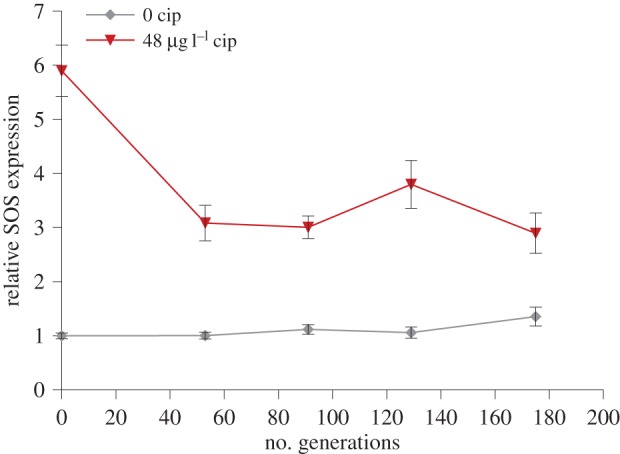
The evolution of SOS response expression. Plotted points show the mean (±s.e.m; *n* = 16) relative expression of a bioluminescent SOS reporter construct in populations of WTpLex : Lux that were allowed to evolve in culture medium lacking ciprofloxacin (grey diamonds) or containing ciprofloxacin (red triangles). We measured the expression of populations under the conditions in which they evolved.

Because the SOS response is regulated by stress, it is conceivable that adaptation to stress will result in altered expression of the SOS pathway. To test this idea, we carried out a second selection experiment in which a WT strain carrying an SOS regulated luminescent reporter construct was allowed to evolve in the presence and absence of ciprofloxacin (figure 4). As expected, the SOS expression of the wild-type strain was substantially increased by exposure to ciprofloxacin at the outset of the experiment (*t*_30_ = 10.20, *p* < 0.001). However, SOS expression rapidly decreased by ≈50% (LM model *t*_78_ = −4.23, *p* < 0.001; figure 4). By contrast, the expression of the SOS response reporter increased marginally (LM model *t*_78_ = 2.31, *p* = 0.024) through time in populations that evolved in the absence of ciprofloxacin (figure 4).

## 4. Discussion

The simplest possible explanation for the evolution of stress–response pathways is that they evolve as a result of direct selection for increased survival or competitive ability under stress. Consistent with this hypothesis, we found that the SOS pathway generates a large benefit, expressed in terms of increased competitive ability, under a sub-MIC dose of ciprofloxacin (figure 2). The strong association between stress–response pathways and elevated mutation rates raises the tantalizing suggestion that natural selection drives the evolution of mechanisms that couple the evolutionary demand for innovation imposed by stress to the supply of evolutionary novelty provided by mutation [4]. Populations derived from both the WT and LexA strains rapidly adapted to ciprofloxacin-containing culture medium by increasing competitive fitness and evolving high levels of ciprofloxacin resistance that are above the current *P. aeruginosa* clinical breakpoint (MIC = 1000 µg l^−1^; EUCAST v. 5.0 Jan, 2015). However, we found no evidence that the SOS pathway increases the evolution of fitness or ciprofloxacin resistance, which is clearly an important component of fitness. These results clearly show that: (i) selection under sublethal doses of antibiotics can rapidly lead to the evolution of clinically relevant resistant phenotypes [35], and (ii) the SOS pathway is not necessary to evolve high levels of fluoroquinolone resistance.

Previous work has shown that the relationship between the mutation supply rate and the rate of adaptation in asexual bacterial populations is nonlinear [36,37]. When the mutation supply rate is low (i.e. when N_e_*u < 1) the rate of adaptation is limited by the rate of appearance of beneficial mutations and increasing the mutation rate accelerates the rate of adaptation. In populations with a high mutation supply rate (i.e. when N_e_*u ≫ 1), increasing the rate of appearance of beneficial mutations has little, if any, impact on the rate of adaptation because competition between beneficial mutations (clonal interference) prevents the fixation of weakly beneficial mutations. We would, therefore, expect that the SOS pathway should only have an impact on evolvability in populations that are mutation limited. However, we estimate that N_e_*u at the outset of our experiment was 0.62 resistance mutations generation^−1^ for WT populations and 0.43 for LexA populations, suggesting that our experiment was carried out in mutation-limited populations.

In mutation-limited populations, the rate of adaptation depends on both the rate at which beneficial mutations arise *de novo* and the rate at which beneficial mutations escape stochastic drift when they are rare. Importantly, the likelihood that a beneficial mutation escapes stochastic loss is proportional to the benefit it confers [38,39]. Although the SOS pathway increases the mutation rate under stress, we argue that the SOS pathway is likely to decrease the fitness benefits associated with beneficial mutations that increase ciprofloxacin resistance, essentially because the SOS pathway maintains the competitive ability of antibiotic sensitive cells. For example, a mutation that confers complete resistance to the dose of ciprofloxacin used in our experiment would be associated with a larger benefit in the LexA mutant strain than in the WT strain, because carrying a functional SOS pathway would no longer provide any additional benefit in ciprofloxacin resistant genotypes. In other words, we argue that the SOS pathway fails to accelerate adaptation under sublethal doses of antibiotic because the small increase in the supply of resistance mutations provided by SOS mutagenesis is at least partially offset by a decrease in the efficacy of selection for elevated resistance. A key prediction of this hypothesis is that the impact of stress-induced mutagenesis on evolvability should be dose-dependent. For example, under lethal doses of antibiotic (i.e. >MIC), any mutation that increases resistance effectively has a selection coefficient of infinity. In populations with a low mutation supply rate, any mechanism that increases the mutation rate, such as SOS mutagenesis, must therefore accelerate evolution. Consistent with this idea, Cirz *et al.* [13] found that the SOS pathway increases the rate of adaptation to lethal doses of ciprofloxacin and rifampicin in a mouse model of *E. coli* infection.

Adaptation to antibiotics usually occurs via three principle mechanisms: altering cellular targets of antibiotics, chemically inactivating antibiotics, or preventing antibiotics from reaching their targets by altered intake or efflux [40–42]. All of these mechanisms are expected to result in a decrease in antibiotic-induced damage, suggesting that evolving resistance to antibiotics should be associated with a decrease in antibiotic-induced stress. For example, we found that rapid adaptation to ciprofloxacin coincided with a decrease in the expression of the SOS pathway (figure 4), and we speculate that the expression of other forms of stress-induced mutagenesis, such as the error-prone polymerase *dinB*, are also likely to have decreased during the experiment in both WT and LexA populations. In any case, these data demonstrate that SOS-associated mutagenesis is likely to be transient over evolutionary time scales, and this may further help to explain why the SOS pathway fails to accelerate long-term adaptation. Alternatively, it is possible that we failed to detect reduced evolvability in populations that were initiated with the LexA mutant because these populations rapidly re-evolved the ability to express the SOS pathway. Recovering the ability to express the SOS pathway would have been associated with a small fitness benefit at the outset of the experiment, on the order of a 7% increase in fitness (figure 2). However, LexA mutant populations rapidly adapted to ciprofloxacin, with an increase in fitness of approximately 30% during the first 50 generations of the experiment (figure 3*b*). This rapid increase in fitness suggests that these populations adapted by first evolving high levels of ciprofloxacin resistance. Given that adaptation to ciprofloxacin is associated with decreased SOS pathway expression in WT populations (figure 4) then selection for recovery of SOS expression in LexA mutant populations is likely to have been short-lived. Furthermore, whole genome sequencing of 15 independently evolved clones derived from the LexA ancestor has failed to uncover any mutations in LexA or any LexA regulated genes (C. Torres-Barcelo, M Toll-Riera, R. Moxon, R. C. Maclean 2014, unpublished data).

In conclusion, our study shows that the SOS pathway increases bacterial fitness, and not evolvability, under sublethal doses of antibiotic. At a fundamental level, these results suggest that the mutagenic effects associated with the SOS pathway are an unwanted side effect, and not a selected attribute, of this pathway. It is important to note that the SOS pathway could increase bacterial evolvability through alternative mechanisms, such as increased horizontal transfer of integrating conjugative elements [43] or the rearrangement of integrons [44]. Several studies have proposed that therapeutic agents which block the SOS response could prove to be important adjuvants that could dramatically increase the efficacy of antibiotics [13,45]. Our results suggest that this strategy will increase the short-term efficacy of antibiotic treatment, but they also suggest that the claim that blocking the SOS will reduce resistance evolution in the long-term should be treated with caution, at least in cases where the evolution of resistance is primarily driven by mutation.

## Supplementary Material

Supplementary Matherial and Methods

## Supplementary Material

Supplementary Figures

## Data accessibility

Data are available at the Dryad repository: http://dx.doi.org/10.5061/dryad.rv766.
