# Supplementary material for: The SOS response increases bacterial fitness, but not evolvability, under a sublethal dose of antibiotic
Source: Proc Biol Sci. 2015 Oct 7;282(1816):20150885. doi: 10.1098/rspb.2015.0885 (PMC4614765; doi:10.1098/rspb.2015.0885)
Supplement: Supplementary Matherial and Methods [file rspb20150885supp1.doc]

**Supplementary Material and Methods**

Generation of WT-YFP strain

To generate a reference strain for competition experiments, we inserted a YFP marker under the control of a constitutive promoter at the *att*Tn7 insertion site of PAO1 using the *Cre-lox* system [1] to generate strain WT-YFP. To study the expression of the SOS response, we constructed the strain WT-pLex-Lux in which we integrated the luminescence operon *luxCDABE* under the control of the *lexA* promoter at the *att*Tn7 chromosomal insertion site of the WT strain. To construct this strain, about 400 base pairs upstream of the *lexA* gene were PCR-amplified using the forward primer 5’- CATATCGGATCCGTCGTCGCTCTGTGTTTTTAT - 3’ (containing a *BamHI* site) and the reverse primer 5’ - CATATCACTAGTCGCCTTCTTCGAACCGAAGTGA - 3’ (containing a *SpeI* site). This promoter region was cloned by using the *BamHI* and *SpeI* sites upstream of a promoter-less *luxCDABE* operon in the pUC18-mini-Tn7T-Gm-*lux* plasmid (accession number: AY962893, [2]). Following the protocol designed by Choi and Schweizer, the resulting plasmid was used to insert the *luxCDABE* operon under the control of the *lexA* promoter at the chromosomal *att*Tn7 site of the WT *P. aeruginosa* strain [2].

Impact of ciprofloxacin on bacterial population growth

To measure the impact on ciprofloxacin on bacterial population growth, we carried out the following experiment. First, we streaked out a glycerol stock of the WT strain on M9KB agar plates. 27 independent colonies were then inoculated into 200uL of M9KB broth and allowed to grow overnight. We then diluted overnight cultures 100 fold into 200 μL of fresh medium containing M9KB supplemented with ciprofloxacin between 0 and 72 μg/L. After overnight incubation, we measured the viable cell titre of diluted samples of bacterial cultures using Bactiter-Glo assays in opaque 96-well plates, as outlined below. To confirm the accuracy of this method, we plated out samples of 3 replicate cultures from the 0 and 48 μg/L of ciprofloxacin treatments on M9KB agar plates and counted CFUs. The relative titres obtained via the two methods were approximately equivalent.

Viable cell titre of cultures using Bactitre-Glo

Bactiter-Glo was added to samples using an on-board automated reagent injector in the plate reader and luminescence measurements were recorded over a 15 minute incubation period at room temperature. We estimated viable cell titre by determining the maximum value of luminescence during the 15 minute incubation for each culture. All luminescence values for both the SOS and Bactiter-Glo assays fell within the linear range of a standard curve that was based on a dilution series of a pooled sample of cultures that were grown at 72 μg/L of ciprofloxacin, and luminescence assays were carried out using opaque 96 well microtiter plates. To confirm the accuracy of the Bactiter-Glo method, we diluted down and plated out samples of the WT and WTpLex:Lux strains that were cultured at 0 and 48 μg/L ciprofloxacin and estimated viable cell density by CFU counts. We found that the relative titre of cultures measured using the two methods was close to identical (not shown).

Estimating fitness by flow cytometry

To determine fitness, we measured changes in the density of the ‘competitor’ strain (WT or LexA) relative to the ‘tester’ strain (WT-YFP) in each population at each growth cycle by flow cytometry. To discriminate between fluorescently labelled and unlabelled cells in mixed populations, we used a BD Accuri™ C6 flow cytometer and software. We recorded 10,000 events per sample, at 1.000 events/µL average flow rate, using fast flow rates (66 μL/min, 22-μm core size) and the thresholds SSC < 8.000 and FSC < 10.000. First, we gated the events based on cell size and complexity (FSC and SSC) to minimize doublets, larger clumps and debris background (Supplementary Figure 3). We estimated the proportion of YFP-tagged and untagged cells in mixed populations of bacteria by setting gates based on the fluorescence emission (FL1: 533/30 nm) of pure cultures of the WT, LexA and WT-YFP strains. We observed no difference in the endogenous autofluorescence of the WT and LexA strains, and the overlap between the distribution of fluorescence intensities of individual cells for the tagged and untagged strains was generally on the order of 9-18%. We corrected for ‘spill-over effect’ in mixed cultures using the method outlined in [3]. To calculate fitness differences between strains, we calculated the number of population doublings of a competitor strain relative to the WT-YFP tester strain, as described in reference [4].

Contamination checks of the long term evolution populations

To check for contamination, we sequenced the *lexA* and gentamicin resistance cassette in all 40 of the populations used for fitness assays. Populations samples of genomic DNA were amplified with the primers Lex_Seq_F (5' GCCACGCCCCGCTCACTGTA 3'), which is complementary to *lexA* and Lex_Seq_R (5' GCGCCGTTGCTGGCCCAGAA 3'), which is complementary to the gentamicin resistance cassette inserted downstream of *lexA* in both the WT and LexA strains. All populations produced amplicons, and sequencing revealed that (i) all populations carried the gentamicin resistance cassette, and (ii) the *lexA* S125A mutation was found in all LexA populations and no WT populations.

**Supplementary Material and Methods references**

1. Mandsberg LF, [Maciá MD](http://www.ncbi.nlm.nih.gov/pubmed?term=Maci∑ MD%5BAuthor%5D&cauthor=true&cauthor_uid=22092761), [Bergmann KR](http://www.ncbi.nlm.nih.gov/pubmed?term=Bergmann KR%5BAuthor%5D&cauthor=true&cauthor_uid=22092761), [Christiansen LE](http://www.ncbi.nlm.nih.gov/pubmed?term=Christiansen LE%5BAuthor%5D&cauthor=true&cauthor_uid=22092761), [Alhede M](http://www.ncbi.nlm.nih.gov/pubmed?term=Alhede M%5BAuthor%5D&cauthor=true&cauthor_uid=22092761), [Kirkby N](http://www.ncbi.nlm.nih.gov/pubmed?term=Kirkby N%5BAuthor%5D&cauthor=true&cauthor_uid=22092761), [Høiby N](http://www.ncbi.nlm.nih.gov/pubmed?term=H¯iby N%5BAuthor%5D&cauthor=true&cauthor_uid=22092761), [Oliver A](http://www.ncbi.nlm.nih.gov/pubmed?term=Oliver A%5BAuthor%5D&cauthor=true&cauthor_uid=22092761), [Ciofu O](http://www.ncbi.nlm.nih.gov/pubmed?term=Ciofu O%5BAuthor%5D&cauthor=true&cauthor_uid=22092761). 2011. Development of antibiotic resistance and up-regulation of the antimutator gene pfpI in mutator *Pseudomonas aeruginosa* due to inactivation of two DNA oxidative repair genes (mutY, mutM). FEMS Microbiol Lett. 324:28-37.
2. Choi K-H, Schweizer HP. 2006. mini-Tn*7* insertion in bacteria with single *att*Tn*7* sites: example *Pseudomonas aeruginosa.* Nat Protoc. 1: 53-161.
3. Heilbron K, Toll-Riera M, Kojadinovic M, MacLean RC. 2014. Fitness is Strongly influenced by rare mutations of large effect in a microbial mutation accumulation experiment. Genetics. 127:981-990.
4. Lenski RE, Rose MR, Simpson SC, Tadler SC. 1991. Long-term experimental evolution in *Escherichia coli* 1. Adaptation and divergence during 2,000 generations. Am Nat. 138:1315-1341.
