# Supplementary material for: The SOS response increases bacterial fitness, but not evolvability, under a sublethal dose of antibiotic
Source: Proc Biol Sci. 2015 Oct 7;282(1816):20150885. doi: 10.1098/rspb.2015.0885 (PMC4614765; doi:10.1098/rspb.2015.0885)
Supplement: Supplementary Figures [file rspb20150885supp2.pdf]

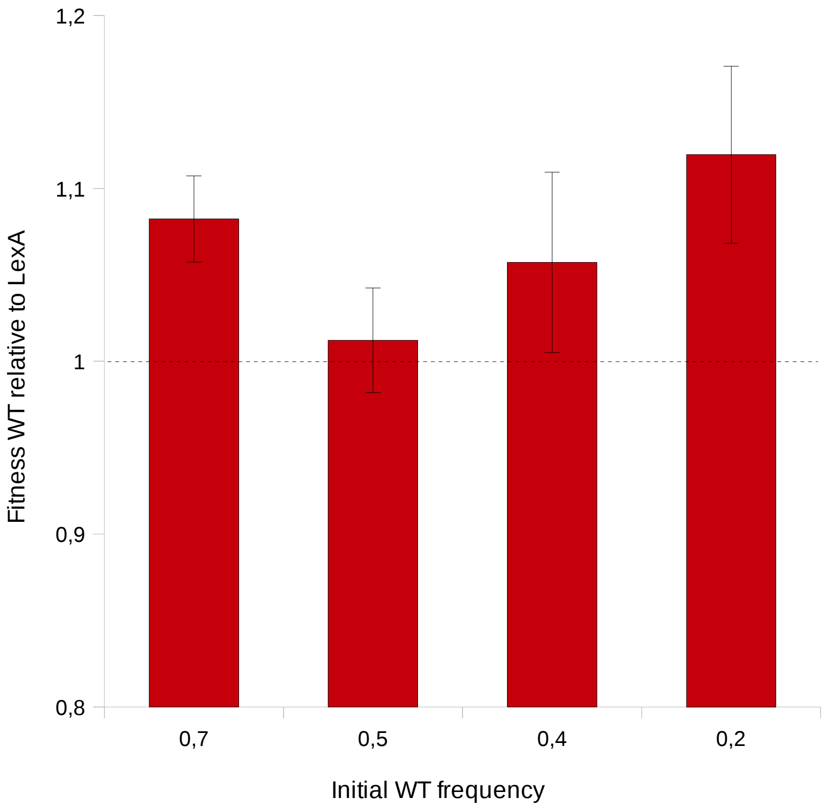

Supplementary Figure 1. Short-term fitness effects of the SOS response. Competitive fitness of the WT strain relative to the LexA mutant, as measured using overnight competition experiments in the presence of 48 µg/L of ciprofloxacin. Values are the average of 5 independent replicates starting from different WT:LexA frequencies and error bars are SEM. The dashed line represents no relative fitness difference (value of 1).

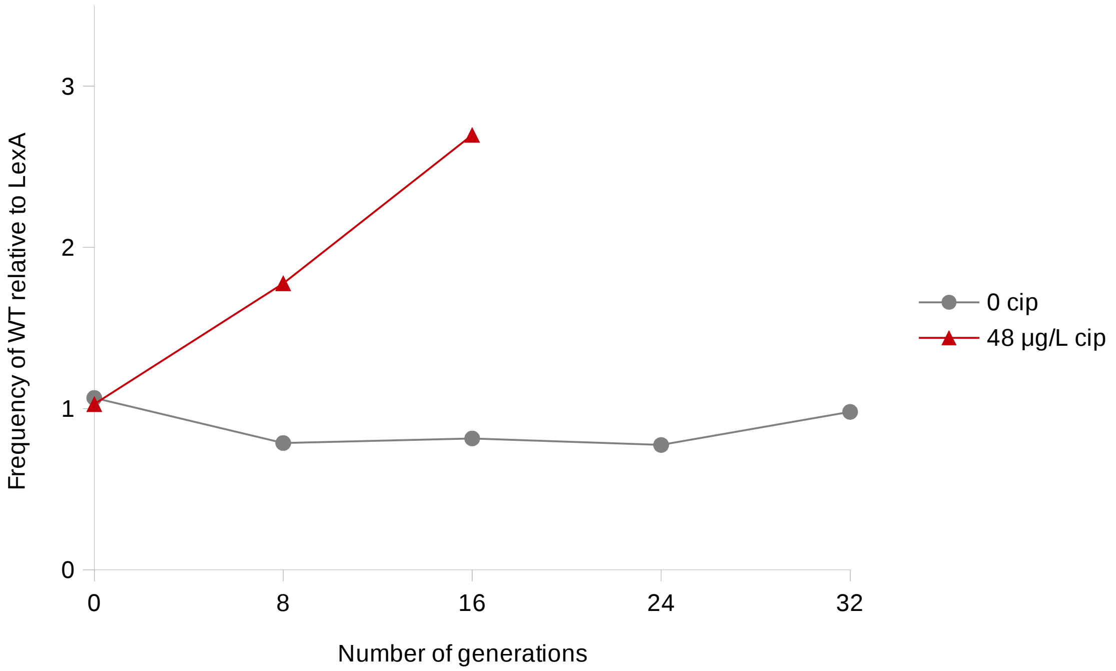

<u>Supplementary Figure 2.</u> <u>Short-term fitness effects of the SOS response.</u> Panel B shows the frequency of the WT strain in competition with the LexA mutant across multiple cycles of competition, such that each day corresponds to 8 generations. After 16 generations, the WT strain had achieved such a high frequency that it could no longer be accurately measured. Values are the mean of six independent replicates and error bars correspond to SEM.

A)

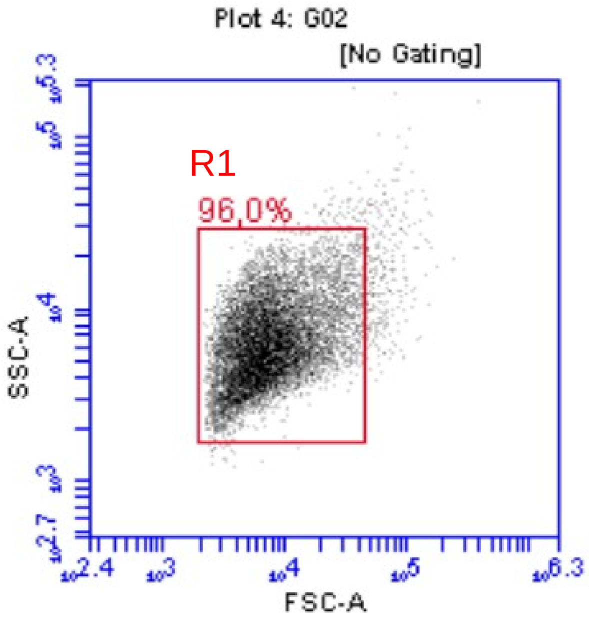

B)

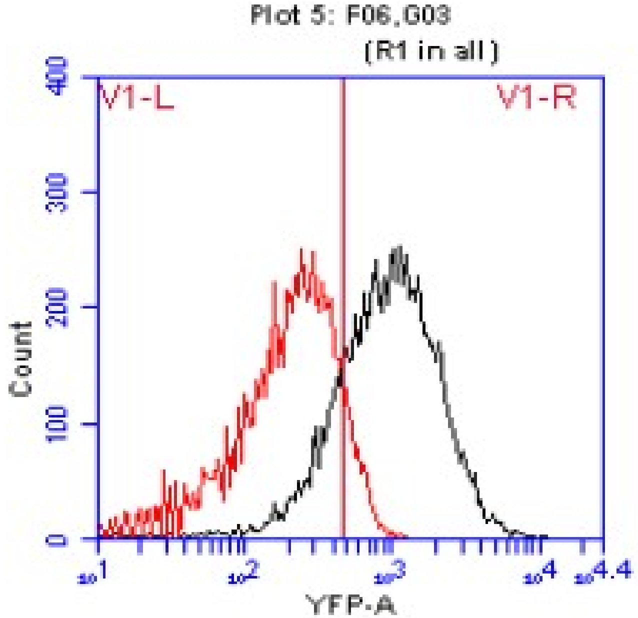

C)

Plot 5: Multiple Samples Gated on (R1 in all)	Count	Volume (µL)	% of This Plot
F06 : V1-L (1,0 / 462,0)	1.741	21,5	18,26%
F06 : V1-R (462,0 / 16.777...	7.793	21,5	81,74%
F06 : This Plot	9.534	21,5	100,00%
G03 : V1-L (1,0 / 462,0)	8.808	16,9	90,89%
G03 : V1-R (462,0 / 16.777...	883	16,9	9,11%
G03 : This Plot	9.691	16,9	100,00%

Supplementary Figure 3. Example of fitness gating in flow cytometry. Examples of FSC-SSC gating (Panel A), and pure culture spil-over of WT and WT-YFP strains (Panel B) showing the percentages of tagged (V1-R) and untagged (V1-L) overlapping used in the correction formula (Table Panel C). In this case: 18.26% of the tagged pure culture spil-over the non YFP fluorescence range (V1-L) and 9.11% of the untagged strain spil-over in the YFP range (V1-R).
